# Supplementary material for: Evaluating the feasibility, fidelity, and preliminary effectiveness of a school-based intervention to improve the school participation and feelings of connectedness of elementary school students on the autism spectrum
Source: PLoS One. 2022 Jun 1;17(6):e0269098. doi: 10.1371/journal.pone.0269098 (PMC9159612; doi:10.1371/journal.pone.0269098)
Supplement: S1 Table — (DOCX) [file pone.0269098.s001.docx]

**S1 Table. Intervention fidelity protocol.**

| **Theoretical element** | **Operational element in the In My Shoes pilot** |
| --- | --- |
| **Study design** | - Intervention is manualised with detailed lesson plans and resources. - Recommended that more than one staff member at each school complete professional learning and familiarise themselves with the In My Shoes program in case of teacher absence. - Recommended dosage (i.e., at least 45 minutes per week over 10 weeks). - Researcher observed delivery of intervention across schools through observation or via video-taped observations. |
| **Training providers** | - Standardised online professional learning video presentations - Pre and post professional learning questionnaires to evaluate teacher confidence in delivering In My Shoes program. - Face to face meeting with implementing teacher and supporting leadership staff to provide opportunity to clarify content of professional learning, answer questions and discuss application of intervention to their classroom. |
| **Delivery of treatment** | - Researcher plans to observe delivery of program across schools through observation or via video-taped observations - Weekly online teacher report fidelity checklist via Qualtrics - Weekly/ fortnightly phone or email check-ins and reminders for teachers to answer questions or provide support |
| **Receipt of treatment** | - In My Shoes situation-based questionnaire to evaluate changes in understanding of content of program - Qualitative evaluation of participant experiences via semi-structured interviews. |
| **Enactment of treatment skills** | - Battery of pre-post outcome measures - Qualitative evaluation of participant experiences via semi-structured interviews. |
