# Supplementary material for: Evaluating the feasibility, fidelity, and preliminary effectiveness of a school-based intervention to improve the school participation and feelings of connectedness of elementary school students on the autism spectrum
Source: PLoS One. 2022 Jun 1;17(6):e0269098. doi: 10.1371/journal.pone.0269098 (PMC9159612; doi:10.1371/journal.pone.0269098)
Supplement: S2 Table — (DOCX) [file pone.0269098.s002.docx]

**S2 Table. Teacher, school leadership and parent responses to anonymous post intervention feedback survey.**

|  | **Response (%)** | | | | |
| --- | --- | --- | --- | --- | --- |
| **Question** | **SD** | **D** | **N** | **A** | **SA** |
| **Teachers (n=8)** | | | | | |
| In My Shoes was a positive experience for me | 0 | 0 | 12.5 | 62.5 | 25 |
| In My Shoes was a positive experience for my classroom | 0 | 0 | 0 | 75 | 25 |
| The content of In My Shoes was relevant in supporting the school participation of students with autism in my classroom | 0 | 0 | 0 | 87.5 | 12.5 |
| The content of In My Shoes was relevant in supporting the school connectedness of students with autism in my classroom | 0 | 0 | 25 | 50 | 25 |
| The content of In My Shoes was important in supporting the school participation of students with autism in my classroom | 0 | 0 | 12.5 | 62.5 | 25 |
| The content of In My Shoes was important in supporting the school connectedness of students with autism in my classroom | 0 | 0 | 12.5 | 75 | 12.5 |
| The outcomes of In My Shoes were beneficial to students with autism in my classroom | 0 | 0 | 0 | 62.5 | 37.5 |
| The outcomes of In My Shoes were beneficial to peers of students with autism in my classroom | 0 | 0 | 0 | 50 | 50 |
| The outcomes of In My Shoes were beneficial to my school as a whole | 0 | 0 | 62.5 | 37.5 | 0 |
| In My Shoes has made sustainable change to the school participation of students with autism in my classroom | 0 | 12.5 | 37.5 | 50 | 0 |
| In My Shoes has made sustainable change to the school connectedness of students with autism in my classroom | 0 | 12.5 | 25 | 62.5 | 0 |
| I would recommend In My Shoes to other schools and/or teachers | 0 | 0 | 12.5 | 50 | 37.5 |
| **School Leadership (e.g., Deputy Principal, Learning Support Coordinator) (n=4)** | | | | | |
| In My Shoes was a positive experience for my school | 0 | 0 | 0 | 50 | 50 |
| The content of In My Shoes was relevant in supporting the school participation of students with autism in the participating classroom | 0 | 0 | 0 | 50 | 50 |
| The content of In My Shoes was relevant in supporting the school connectedness of students with autism in the participating classroom | 0 | 0 | 0 | 50 | 50 |
| The content of In My Shoes was important in supporting the school participation of students with autism in the participating classroom. | 0 | 0% | 0 | 100 | 0 |
| The content of In My Shoes was important in supporting the school connectedness of students with autism in the participating classroom. | 0 | 0 | 0 | 50 | 50 |
| The outcomes of In My Shoes were beneficial to students with autism in the participating classroom | 0 | 0 | 0 | 75 | 25 |
| The outcomes of In My Shoes were beneficial to peers in the participating classroom | 0 | 0 | 0 | 25 | 75 |
| The outcomes of In My Shoes were beneficial to teachers in the participating classroom/s | 0 | 0 | 0 | 50 | 50 |
| The outcomes of In My Shoes were beneficial to my school as a whole | 0 | 0 | 75 | 0 | 25 |
| In My Shoes has made sustainable change to the school participation of students with autism in the participating classroom | 0 | 0 | 50 | 50 | 0 |
| In My Shoes has made sustainable change to the school connectedness of students with autism in the participating classroom | 0 | 0 | 25 | 50 | 25 |
| I would recommend In My Shoes to other schools | 0 | 0 | 0 | 25 | 75 |
| **Parents (n=10)** | | | | | |
| In My Shoes was a positive experience for me and my child | 0 | 0 | 30 | 50 | 20 |
| The content of In My Shoes is relevant in supporting the school participation of students with autism in mainstream schools | 0 | 0 | 10 | 60 | 30 |
| The content of In My Shoes is relevant in supporting the school connectedness of students with autism in mainstream schools | 0 | 0 | 10 | 60 | 30 |
| The content of In My Shoes is important in supporting the school participation of students with autism in mainstream schools | 0 | 0 | 10 | 60 | 30 |
| The content of In My Shoes is important in supporting the school connectedness of students with autism in mainstream schools | 0 | 0 | 10 | 60 | 30 |
| The outcomes of In My Shoes were beneficial to my child | 0 | 0 | 30 | 40 | 20 |
| The parent involvement required in In My Shoes was manageable (e.g., reading weekly parent information handouts; trying to incorporate suggested strategies at home; attending/participating in Module 10). | 0 | 0 | 50 | 40 | 10 |
| In My Shoes has made sustainable change for the school participation of my child | 0 | 0 | 50 | 40 | 10 |
| In My Shoes has made sustainable change for the school connectedness of my child | 0 | 10 | 0 | 60 | 30 |
| I would recommend In My Shoes to other parents or school | 0 | 0 | 10 | 60 | 30 |
| *Notes.* SD: Strongly Disagree; D: Disagree; N: Neutral; A: Agree; SA: Strongly Agree. | | | | | |
