# Supplementary material for: Evaluating the feasibility, fidelity, and preliminary effectiveness of a school-based intervention to improve the school participation and feelings of connectedness of elementary school students on the autism spectrum
Source: PLoS One. 2022 Jun 1;17(6):e0269098. doi: 10.1371/journal.pone.0269098 (PMC9159612; doi:10.1371/journal.pone.0269098)
Supplement: S3 Table — (DOCX) [file pone.0269098.s003.docx]

**S3 Table. Student responses to anonymous paper-based post intervention feedback survey**

| Students (n=200) | **Response (%)** | | | |
| --- | --- | --- | --- | --- |
| **Question** | **SD** | **D** | **A** | **SA** |
| *In My Shoes* was fun | 4.5 | 10.5 | 56.0 | 29.0 |
| I enjoyed *In My Shoes* | 5.5 | 12.0 | 51.0 | 31.5 |
| *In My Shoes* activities were interesting; | 4.0 | 10.5 | 56.0 | 29.5 |
| *In My Shoes* activities made sense to me; | 4.0 | 12.5 | 55.0 | 28.5 |
| *In My Shoes* activities were easy to do; | 6.5 | 18.0 | 47.5 | 28.0 |
| The lessons taught in *In My Shoes* are important; | 3.0 | 3.5 | 33.0 | 60.5 |
| I learnt something new from *In My Shoes*; | 4.0 | 12.0 | 38.0 | 46.0 |
| Notes. SD= strongly disagree; D= disagree; A= agree; SA= strongly agree | | | | |
