# Supplementary material for: Evaluating the feasibility, fidelity, and preliminary effectiveness of a school-based intervention to improve the school participation and feelings of connectedness of elementary school students on the autism spectrum
Source: PLoS One. 2022 Jun 1;17(6):e0269098. doi: 10.1371/journal.pone.0269098 (PMC9159612; doi:10.1371/journal.pone.0269098)
Supplement: S4 Table — (DOCX) [file pone.0269098.s004.docx]

**S4 Table. Difference in objective ESM data pre-post intervention, autism sample**

| **n=10** |  | **Pre Mean**  **% of time (SD)** | **Post Mean**  **% of time (SD)** | **Z score** | **P value** |
| --- | --- | --- | --- | --- | --- |
| **Where were you?** | Classroom | 40.10 (15.45) | 43.50 (16.68) | 0.663 | 0.507 |
|  | At home or on way to/from home/school | 26.70 (9.04) | 28.70 (12.32) | 0.474 | 0.635 |
|  | Outside classroom | 21.70 (13.59) | 15.80 (9.41) | 1.686 | 0.092 |
|  | Specialist subject | 8.20 (13.65) | 6.90 (6.15) | 0.338 | 0.735 |
|  | Other | 3.40 (4.42) | 5.10 (7.40) | 0.315 | 0.752 |
| **What was the main thing you were doing?** | Listening to teacher | 28.50 (18.55) | 22.40 (11.19) | 1.125 | 0.261 |
|  | Classwork – incl. homework, individual and small group work | 19.40 (15.33) | 18.00 (9.92) | 0.415 | 0.678 |
|  | Leisure – incl. physical activity, ipad, gaming, resting, seeing friends and family and reading | 18.30 (13.05) | 18.70 (11.18) | 0.306 | 0.759 |
|  | Transition | 13.00 (17.61) | 11.80 (11.95) | 0.297 | 0.766 |
|  | Self-care – incl. eating, dressing, toileting | 10.60 (8.39) | 10.40 (7.84) | 0.060 | 0.853 |
|  | Play | 8.90 (10.52) | 13.20 (13.87) | 0.889 | 0.374 |
|  | Something else | 1.10 (3.47) | 5.80 (7.00) | 2.032 | 0.042* |
| **Who were you with?** | Teacher | 20.50 (13.06) | 22.80 (15.25) | 0.890 | 0.373 |
|  | EA | 12.40 (12.77) | 7.40 (10.28) | 1.400 | 0.161 |
|  | Classmate | 42.50 (9.05) | 50.20 (20.91) | 0.918 | 0.359 |
|  | Family | 26.80 (11.28) | 26.20 (13.61) | 0.051 | 0.959 |
|  | Alone | 9.50 (14.33) | 8.10 (10.84) | 0.140 | 0.889 |
|  | Someone else – e.g., neighbour, doctor | 2.40 (3.86) | 6.90 (6.55) | 1.628 | 0.103 |
| **Would you prefer to be alone?** | Yes | 30.90 (31.07) | 33.50 (27.71) | 0.415 | 0.678 |
| **Were you talking with someone?** | Yes | 39.10 (22.62) | 39.10 (22.62) | 0.408 | 0.683 |
|  | No | 62.60 (24.28) | 61.00 (22.58) | 0.357 | 0.721 |
| **Who were you talking to?** | Teacher | 2.30 (5.12) | 5.40 (6.29) | 1.214 | 0.225 |
|  | EA | 5.80 (13.89) | 3.00 (5.65) | 0.730 | 0.465 |
|  | Classmate | 21.50 (14.93) | 18.60 (12.03) | 0.663 | 0.507 |
|  | Family | 13.40 (12.69) | 10.90 (13.73) | 0.762 | 0.446 |
|  | Someone else | 2.80 (5.11) | 4.90 (6.06) | 0.931 | 0.352 |
| **Did you need help?** | Yes | 17.50 (17.21) | 11.20 (11.10) | 1.125 | 0.260 |
| **Who helped you?** | Teacher | 15.90 (28.03) | 8.30 (17.95) | 1.069 | 0.285 |
|  | EA | 23.30 (41.71) | 3.30 (10.43) | 1.604 | 0.109 |
|  | Classmate | 5.00 (15.81) | 8.30 (17.95) | 0.272 | 0.785 |
|  | Family | 3.30 (10.43) | 36.70 (42.19) | 2.032 | 0.042* |
|  | Other | 32.50 (44.17) | 13.30 (32.18) | 1.604 | 0.109 |
| **When you were [insert activity], would you prefer to be doing another activity?** | Listening to teacher/ Yes | 31.70 (41.75) | 58.30 (37.86) | 2.213 | 0.027* |
|  | Classwork/ Yes | 28.30 (41.60) | 37.30 (39.12) | 0.962 | 0.336 |
|  | Play/ Yes | 0.00 (0.00) | 11.70 (24.98) | 1.342 | 0.180 |
|  | Transition / Yes | 25.00 (42.49) | 28.30 (34.28) | 0.136 | 0.892 |
|  | Leisure / Yes | 10.00 (21.08) | 22.50 (41.58) | 0.921 | 0.357 |
|  | Self-care / Yes | 30.00 (42.91) | 55.00 (43.78) | 1.633 | 0.102 |
|  | Something else / Yes | 5.00 (15.81) | 10.00 (31.62) | 0.447 | 0.655 |
| **When you were talking, would you prefer to be alone?** | Yes | 10.80 (24.82) | 17.30 (26.86) | 0.730 | 0.465 |
| **When you needed help, who helped you?** | Teacher | 30.00 (48.30) | 20.00 (42.16) | 1.000 | 0.317 |
|  | EA | 25.00 (42.49) | 10.00 (31.62) | 1.089 | 0.276 |
|  | Classmate | 10.00 (31.62) | 20.00 (42.16) | 0.577 | 0.564 |
|  | Family | 10.00 (31.62) | 50.00 (52.70) | 2.000 | 0.046* |
|  | Other | 40.00 (51.64) | 20.00 (42.16) | 1.414 | 0.157 |
| **When you were [insert activity] did you need help?** | Listening to the teacher | 27.50 (32.38) | 19.20 (32.87) | 0.768 | 0.443 |
|  | Classwork | 15.80 (32.00) | 0.00 (0.00) | 1.604 | 0.109 |
|  | Play | 0.00 (0.00) | 2.50 (7.90) | 1.000 | 0.317 |
|  | Transition | 2.50 (7.90) | 16.70 (27.28) | 1.604 | 0.109 |
|  | Leisure | 15.00 (33.74) | 5.00 (15.81) | 0.816 | 0.414 |
|  | Selfcare | 0.00 (0.00) | 5.00 (15.81) | 1.000 | 0.317 |
|  | Something else | 5.00 (15.81) | 0.00 (0.00) | 1.000 | 0.317 |
| Note. *p* <0.05 | | | | | |
