# Supplementary material for: Evaluating the feasibility, fidelity, and preliminary effectiveness of a school-based intervention to improve the school participation and feelings of connectedness of elementary school students on the autism spectrum
Source: PLoS One. 2022 Jun 1;17(6):e0269098. doi: 10.1371/journal.pone.0269098 (PMC9159612; doi:10.1371/journal.pone.0269098)
Supplement: S5 Table — (DOCX) [file pone.0269098.s005.docx]

**S5 Table. Difference in subjective ESM data pre-post intervention, autism sample**

| **n=10** | **Pre**  **Mean (SD)** | **Post**  **Mean (SD)** | **Z score** | **P value** |
| --- | --- | --- | --- | --- |
| **Enjoyment** | 69.30 (11.70) | 71.10 (12.91) | 0.561 | 0.575 |
| **Difficulty** | 21.40 (11.29) | 23.00 (17.16) | 0.459 | 0.646 |
| **Interest** | 65.20 (11.66) | 62.30 (16.41) | 1.173 | 0.241 |
| **Importance** | 61.10 (19.72) | 56.10 (24.97) | 1.123 | 0.262 |
| **Help amount** | 60.75 (29.59) | 41.14 (29.27) | 1.156 | 0.248 |
| **Sad to happy** | 83.60 (15.22) | 80.90 (20.41) | 0.357 | 0.721 |
| **Worried to relaxed** | 86.30 (13.78) | 79.60 (16.77) | 1.378 | 0.168 |
| **Lonely to sociable** | 81.80 (14.84) | 76.50 (16.77) | 1.071 | 0.284 |
| **Bored to excited** | 70.70 (16.15) | 65.40 (20.00) | 0.867 | 0.386 |
| **Angry to calm** | 85.70 (15.88) | 78.00 (19.62) | 1.893 | 0.058 |
| **Enjoyment – Activity** | | | | |
| **Listening to teacher** | 59.11 (22.71) | 61.90 (28.60) | 0.415 | 0.678 |
| **Classwork** | 67.87 (31.83) | 67.00 (32.66) | 0.314 | 0.753 |
| **Play** | 98.60 (2.608) | 89.43 (17.73) | 0.447 | 0.655 |
| **Transitioning** | 55.50 (23.93) | 63.71 (38.76) | 0.365 | 0.715 |
| **Leisure** | 81.37 (28.17) | 87.88 (14.50) | 0.944 | 0.345 |
| **Self-care** | 75.57 (25.36) | 57.12 (39.90) | 2.207 | 0.027* |
| **Something else** | 51.00 (0.00) | 66.60 (31.30) | - | - |
| **Difficulty – Activity** | | | | |
| **Listening to teacher** | 26.67 (37.63) | 32.20 (26.41) | 0.593 | 0.553 |
| **Classwork** | 33.37 (25.19) | 27.88 (22.15) | 1.153 | 0.249 |
| **Play** | 2.60 (2.30) | 27.00 (34.18) | 1.342 | 0.180 |
| **Transitioning** | 16.50 (24.114) | 17.67 (19.88) | 1.000 | 0.317 |
| **Leisure** | 28.87 (35.00) | 13.55 (16.54) | 0.674 | 0.500 |
| **Self-care** | 9.42 (15.03) | 13.12 (20.46) | 0.944 | 0.345 |
| **Something else** | 51.00 (0.00) | 6.80 (14.65) | - | - |
| **Interest – Activity** | | | | |
| **Listening to teacher** | 64.77 (34.92) | 50.70 (29.98) | 1.400 | 0.161 |
| **Classwork** | 58.87 (23.17) | 60.55 (28.80) | 0.000 | 1.000 |
| **Play** | 95.00 (9.59) | 83.71 (24.93) | 0.447 | 0.655 |
| **Transitioning** | 52.83 (34.38) | 61.16 (34.11) | 1.069 | 0.285 |
| **Leisure** | 80.37 (21.13) | 78.77 (17.54) | 0.271 | 0.786 |
| **Self-care** | 53.42 (24.43) | 46.25 (39.08) | 1.992 | 0.046* |
| **Something else** | 16.00 (0.00) | 66.80 (47.02) | - | - |
| **Importance – Activity** | | | | |
| **Listening to teacher** | 64.88 (33.52) | 41.40 (28.83) | 2.310 | 0.021* |
| **Classwork** | 49.87 (31.91) | 48.22 (32.07) | 1.014 | 0.310 |
| **Play** | 69.20 (40.59) | 53.57 (33.75) | 1.604 | 0.109 |
| **Transitioning** | 53.33 (30.29) | 77.50 (24.87) | 1.604 | 0.109 |
| **Leisure** | 73.87 (30.35) | 62.00 (34.41) | 1.992 | 0.046* |
| **Self-care** | 35.85 (36.20) | 77.50 (35.09) | 1.363 | 0.173 |
| **Something else** | 51.00 (0.00) | 75.20 (24.95) | - | - |
| **Enjoyment – Place** | | | | |
| **Classroom** | 56.70 (20.82) | 65.10 (22.90) | 1.531 | 0.126 |
| **Outside classroom** | 64.55 (38.06) | 89.22 (19.56) | 1.014 | 0.310 |
| **Specialist classroom** | 81.80 (19.70) | 47.67 (37.75) | 1.826 | 0.068 |
| **At home transition** | 77.30 (20.70) | 68.60 (24.11) | 0.949 | 0.343 |
| **Other** | 79.50 (20.53) | 85.00 (18.73) | 1.342 | 0.180 |
| **Difficulty – Place** | | | | |
| **Classroom** | 36.40 (23.33) | 24.20 (24.70) | 2.295 | 0.022* |
| **Outside classroom** | 18.88 (32.09) | 23.44 (22.66) | 0.676 | 0.499 |
| **Specialist classroom** | 25.60 (24.66) | 36.67 (31.98) | 1.461 | 0.144 |
| **At home transition** | 16.80 (17.70) | 22.20 (18.69) | 0.771 | 0.441 |
| **Other** | 3.25 (4.50) | 2.00 (1.73) | 0.447 | 0.655 |
| **Interest – Place** | | | | |
| **Classroom** | 58.60 (21.76) | 59.10 (26.22) | 0.051 | 0.959 |
| **Outside classroom** | 71.33 (32.61) | 79.56 (26.72) | 0.169 | 0.866 |
| **Specialist classroom** | 85.00 (22.04) | 40.17 (24.45) | 1.841 | 0.066 |
| **At home transition** | 70.80 (18.15) | 63.00 (31.05) | 0.830 | 0.407 |
| **Other** | 66.00 (44.87) | 57.33 (40.67) | 0.447 | 0.655 |
| **Importance – Place** | | | | |
| **Classroom** | 58.60 (26.18) | 55.90 (27.50) | 0.765 | 0.444 |
| **Outside classroom** | 51.55 (38.34) | 65.33 (36.29) | 0.507 | 0.612 |
| **Specialist classroom** | 64.40 (39.20) | 38.50 (28.19) | 0.730 | 0.465 |
| **At home transition** | 60.80 (23.42) | 62.50 (34.53) | 0.415 | 0.678 |
| **Other** | 52.70 (54.68) | 56.67 (48.33) | 1.000 | 0.317 |
| **Enjoyment – Who with** | | | | |
| **Teacher** | 54.78 (34.72) | 75.56 (20.59) | 1.718 | 0.086 |
| **EA** | 70.00 (34.07) | 54.00 (35.61) | 1.461 | 0.144 |
| **Classmate** | 67.00 (17.21) | 70.00 (21.04) | 0.000 | 1.00 |
| **Family** | 73.22 (15.78) | 71.77 (23.51) | 1.014 | 0.310 |
| **Alone** | 73.40 (25.36) | 78.00 (31.49) | 0.447 | 0.655 |
| **Someone else** | 76.33 (40.99) | 72.50 (18.17) | - | - |
| **Difficulty – Who with** | | | | |
| **Teacher** | 38.00 (28.87) | 25.67 (28.526) | 1.599 | 0.110 |
| **EA** | 31.13 (39.654) | 21.75 (23.991) | 0.535 | 0.593 |
| **Classmate** | 24.60 (21.72) | 24.40 (24.20) | 0.560 | 0.575 |
| **Family** | 11.22 (13.04) | 23.22 (20.12) | 1.103 | 0.270 |
| **Alone** | 15.20 (15.53) | 6.00 (11.18) | 1.342 | 0.180 |
| **Someone else** | 10.66 (8.50) | 18.83 (29.171) | - | - |
| **Interest – Who with** | | | | |
| **Teacher** | 51.33 (36.609) | 59.11 (26.812) | 0.280 | 0.779 |
| **EA** | 74.38 (30.94) | 52.00 (34.90) | 1.461 | 0.144 |
| **Classmate** | 66.50 (17.56) | 61.50 (22.46) | 0.306 | 0.759 |
| **Family** | 64.66 (14.23) | 61.33 (32.88) | 0.631 | 0.528 |
| **Alone** | 43.00 (40.40) | 73.00 (39.71) | 1.342 | 0.180 |
| **Someone else** | 88.33 (20.20) | 64.83 (22.69) | - | - |
| **Talking – Emotions** | | | | |
| **Enjoyment** | 75.88 (12.91) | 68.40 (29.20) | 0.296 | 0.767 |
| **Difficulty** | 22.22 (17.48) | 22.80 (23.13) | 0.421 | 0.674 |
| **Interest** | 72.22 (10.98) | 63.60 (34.28) | 0.770 | 0.441 |
| **Importance** | 68.00 (19.62) | 51.70 (30.43) | 1.255 | 0.209 |
| **Enjoyment – Talking to** | | | | |
| **Teacher** | 92.00 (11.31) | 61.80 (41.09) | 1.00 | 0.317 |
| **EA** | 71.50 (17.67) | 53.33 (49.74) | - | - |
| **Classmate** | 70.667 (21.17) | 78.90 (32.65) | 0.889 | 0.374 |
| **Family** | 65.71 (33.78) | 54.42 (41.02) | 0.943 | 0.345 |
| **Someone else** | 92.67 (12.702) | 72.80 (43.580) | - | - |
| **Difficulty – Talking to** | | | | |
| **Teacher** | 24.50 (33.23) | 35.40 (34.45) | 1.342 | 0.180 |
| **EA** | 31.50 (26.16) | 47.33 (49.803) | - | - |
| **Classmate** | 26.44 (21.48) | 19.20 (27.40) | 0.676 | 0.499 |
| **Family** | 16.85 (36.93) | 17.42 (36.97) | 0.184 | 0.854 |
| **Someone else** | 13.33 (21.362) | 13.40 (28.85) | - | - |
| **Interest – Talking to** | | | | |
| **Teacher** | 75.50 (34.64) | 67.20 (29.25) | 0.000 | 1.000 |
| **EA** | 52.00 (26.87) | 53.67 (49.80) | - | - |
| **Classmate** | 65.88 (16.97) | 67.10 (42.14) | 0.296 | 0.767 |
| **Family** | 60.42 (31.45) | 48.71 (42.27) | 1.214 | 0.225 |
| **Someone else** | 93.33 (11.54) | 63.80 (50.022) | - | - |
| **Listening to teacher – Emotions** | | | | |
| **Sad to happy** | 75.88 (29.97) | 81.30 (26.20) | 0.415 | 0.678 |
| **Worried to relaxed** | 80.77 (32.96) | 76.70 (24.27) | 0.931 | 0.352 |
| **Lonely to sociable** | 72.77 (29.35) | 80.50 (19.78) | 0.593 | 0.553 |
| **Bored to excited** | 58.88 (35.58) | 56.30 (42.42) | 0.280 | 0.779 |
| **Angry to calm** | 78.44 (35.45) | 76.90 (27.64) | 0.561 | 0.575 |
| **Classwork – Emotions** | | | | |
| **Sad to happy** | 85.62 (16.94) | 71.88 (25.85) | 1.625 | 0.104 |
| **Worried to relaxed** | 88.37 (12.05) | 79.88 (21.12) | 2.023 | 0.043* |
| **Lonely to sociable** | 81.00 (14.72) | 76.66 (20.17) | 1.472 | 0.141 |
| **Bored to excited** | 81.00 (15.70) | 66.11 (30.88) | 1.153 | 0.249 |
| **Angry to calm** | 87.37 (14.13) | 73.55 (31.50) | 1.355 | 0.176 |
| **Play – Emotions** | | | | |
| **Sad to happy** | 93.80 (10.40) | 83.43 (28.37) | 0.447 | 0.655 |
| **Worried to relaxed** | 89.00 (15.06) | 86.29 (23.48) | 0.447 | 0.655 |
| **Lonely to sociable** | 89.40 (12.03) | 74.29 (22.19) | 0.447 | 0.655 |
| **Bored to excited** | 91.80 (11.49) | 78.57 (21.44) | 1.342 | 0.180 |
| **Angry to calm** | 96.20 (8.497) | 83.29 (21.80) | 1.342 | 0.180 |
| **Transition – Emotions** | | | | |
| **Sad to happy** | 87.00 (17.11) | 86.16 (19.69) | 0.447 | 0.655 |
| **Worried to relaxed** | 74.17 (37.24) | 82.66 (26.02) | 0.535 | 0.593 |
| **Lonely to sociable** | 72.50 (38.22) | 81.00 (23.57) | 0.000 | 1.000 |
| **Bored to excited** | 61.33 (29.53) | 68.66 (25.05) | 1.069 | 0.285 |
| **Angry to calm** | 74.50 (37.41) | 85.00 (22.61) | 0.535 | 0.593 |
| **Leisure – Emotions** | | | | |
| **Sad to happy** | 90.00 (17.58) | 81.88 (24.23) | 0.674 | 0.500 |
| **Worried to relaxed** | 94.00 (14.69) | 84.11 (18.41) | 0.944 | 0.345 |
| **Lonely to sociable** | 87.37 (18.65) | 76.22 (24.90) | 0.676 | 0.499 |
| **Bored to excited** | 81.62 (25.12) | 66.22 (29.23) | 2.207 | 0.027* |
| **Angry to calm** | 89.37 (19.84) | 80.66 (26.72) | 0.674 | 0.500 |
| **Self-care – Emotions** | | | | |
| **Sad to happy** | 86.60 (12.52) | 80.37 (27.42) | 0.730 | 0.465 |
| **Worried to relaxed** | 85.00 (17.52) | 78.87 (24.60) | 0.674 | 0.500 |
| **Lonely to sociable** | 86.00 (17.46) | 83.37 (21.09) | 1.214 | 0.225 |
| **Bored to excited** | 67.16 (32.71) | 64.50 (25.46) | 0.314 | 0.753 |
| **Angry to calm** | 92.33 (10.67) | 76.00 (35.37) | 1.363 | 0.173 |
| **Something else – Emotions** | | | | |
| **Sad to happy** | 100.00 (0.00) | 73.40 (25.25) | - | - |
| **Worried to relaxed** | 100.00 (0.00) | 77.80 (22.11) | - | - |
| **Lonely to sociable** | 100.00 (0.00) | 80.00 (12.53) | - | - |
| **Bored to excited** | 51.00 (0.00) | 66.80 (35.68) | - | - |
| **Angry to calm** | 100.00 (0.00) | 67.60 (27.05) | - | - |
| **Classroom – Emotions** | | | | |
| **Sad to happy** | 75.50 (28.90) | 77.90 (22.31) | 0.059 | 0.953 |
| **Worried to relaxed** | 83.70 (22.12) | 82.00 (19.83) | 1.244 | 0.214 |
| **Lonely to sociable** | 75.70 (26.95) | 83.20 (17.94) | 1.888 | 0.059 |
| **Bored to excited** | 63.60 (24.85) | 67.70 (29.74) | 0.060 | 0.952 |
| **Angry to calm** | 78.90 (31.01) | 72.80 (28.01) | 1.186 | 0.236 |
| **Outside classroom – Emotions** | | | | |
| **Sad to happy** | 86.44 (18.17) | 88.11 (25.32) | 0.170 | 0.865 |
| **Worried to relaxed** | 90.33 (10.39) | 84.78 (17.59) | 1.524 | 0.128 |
| **Lonely to sociable** | 90.33 (10.93) | 76.44 (23.90) | 1.260 | 0.208 |
| **Bored to excited** | 70.11 (33.90) | 79.22 (23.50) | 0.314 | 0.753 |
| **Angry to calm** | 89.22 (17.39) | 81.22 (26.20) | 1.572 | 0.116 |
| **Specialist – Emotions** | | | | |
| **Sad to happy** | 94.00 (12.00) | 82.17 (24.72) | 1.000 | 0.317 |
| **Worried to relaxed** | 97.50 (5.00) | 79.67 (24.32) | 1.342 | 0.180 |
| **Lonely to sociable** | 88.00 (14.23) | 77.67 (28.79) | - | - |
| **Bored to excited** | 75.25 (49.50) | 33.33 (38.89) | 1.604 | 0.109 |
| **Angry to calm** | 100.00 (0.00) | 88.83 (20.63) | 1.342 | 0.180 |
| **At home or transition to/from school – Emotions** | | | | |
| **Sad to happy** | 88.50 (16.29) | 80.10 (23.00) | 0.816 | 0.415 |
| **Worried to relaxed** | 83.70 (17.65) | 77.50 (22.69) | 0.889 | 0.374 |
| **Lonely to sociable** | 84.80 (19.66) | 72.10 (18.63) | 1.244 | 0.214 |
| **Bored to excited** | 77.30 (22.70) | 62.60 (19.54) | 1.480 | 0.139 |
| **Angry to calm** | 86.70 (14.84) | 79.60 (28.16) | 0.415 | 0.678 |
| **Other – Emotions** | | | | |
| **Sad to happy** | 81.50 (24.22) | 82.00 (15.71) | 0.447 | 0.655 |
| **Worried to relaxed** | 94.25 (11.50) | 78.66 (18.90) | 0.447 | 0.655 |
| **Lonely to sociable** | 83.75 (22.18) | 80.33 (24.41) | 0.447 | 0.655 |
| **Bored to excited** | 75.00 (26.94) | 76.66 (20.30) | 0.447 | 0.655 |
| **Angry to calm** | 93.50 (13.50) | 50.66 (44.79) | 1.342 | 0.180 |
| **Who Teacher – Emotions** | | | | |
| **Sad to happy** | 71.56 (33.72) | 89.33 (17.42) | 1.400 | 0.161 |
| **Worried to relaxed** | 88.44 (19.12) | 74.22 (21.99) | 1.823 | 0.068 |
| **Lonely to sociable** | 72.11 (35.39) | 80.22 (20.88) | 0.423 | 0.672 |
| **Bored to excited** | 65.67 (35.37) | 60.11 (25.50) | 0.140 | 0.889 |
| **Angry to calm** | 77.44 (34.15) | 76.67 (32.02) | 0.339 | 0.735 |
| **Who EA – Emotions** | | | | |
| **Sad to happy** | 84.00 (23.58) | 93.75 (11.84) | 0.000 | 1.000 |
| **Worried to relaxed** | 94.63 (10.12) | 74.75 (19.77) | 1.826 | 0.068 |
| **Lonely to sociable** | 83.13 (24.85) | 80.50 (13.17) | 1.461 | 0.144 |
| **Bored to excited** | 79.25 (24.30) | 56.00 (41.38) | 1.289 | 0.197 |
| **Angry to calm** | 94.88 (9.93) | 75.75 (19.25) | 1.826 | 0.068 |
| **Who Classmate – Emotions** | | | | |
| **Sad to happy** | 85.00 (15.83) | 80.00 (21.29) | 0.765 | 0.444 |
| **Worried to relaxed** | 87.80 (16.71) | 84.40 (17.65) | 1.224 | 0.221 |
| **Lonely to sociable** | 84.20 (16.20) | 78.30 (20.33) | 0.818 | 0.413 |
| **Bored to excited** | 69.90 (2037) | 62.60 (30.42) | 1.070 | 0.285 |
| **Angry to calm** | 86.70 (16.85) | 75.20 (21.92) | 2.552 | 0.011* |
| **Who Family – Emotions** | | | | |
| **Sad to happy** | 88.44 (15.33) | 81.33 (24.33) | 0.943 | 0.345 |
| **Worried to relaxed** | 86.88 (13.43) | 80.88 (23.43) | 1.367 | 0.172 |
| **Lonely to sociable** | 85.77 (14.40) | 72.66 (18.11) | 1.352 | 0.176 |
| **Bored to excited** | 75.55 (22.66) | 67.33 (21.55) | 0.593 | 0.553 |
| **Angry to calm** | 88.44 (12.54) | 79.11 (24.83) | 1.609 | 0.108 |
| **Who Alone – Emotions** | | | | |
| **Sad to happy** | 82.20 (24.06) | 84.60 (22.13) | 1.000 | 0.317 |
| **Worried to relaxed** | 90.00 (20.19) | 77.60 (30.68) | 1.000 | 0.317 |
| **Lonely to sociable** | 82.40 (22.25) | 85.00 (23.28) | 1.000 | 0.317 |
| **Bored to excited** | 66.80 (45.48) | 66.00 (23.52) | 1.000 | 0.317 |
| **Angry to calm** | 91.20 (17.52) | 71.20 (29.01) | 1.000 | 0.317 |
| **Who Someone else – Emotions** | | | | |
| **Sad to happy** | 80.00 (28.28) | 83.00 (18.665) | - | - |
| **Worried to relaxed** | 92.50 (10.60) | 78.67 (18.55) | - | - |
| **Lonely to sociable** | 92.00 (11.31) | 74.33 (16.35) | - | - |
| **Bored to excited** | 61.500 (54.44) | 66.83 (25.63) | - | - |
| **Angry to calm** | 85.00 (21.21) | 91.67 (20.41) | - | - |
| **Talking – Emotions** | | | | |
| **Sad to happy** | 86.66 (7.72) | 79.00 (28.72) | 0.830 | 0.407 |
| **Worried to relaxed** | 88.33 (11.06) | 79.10 (20.92) | 1.400 | 0.161 |
| **Lonely to sociable** | 87.88 (8.38) | 79.20 (21.57) | 1.120 | 0.263 |
| **Bored to excited** | 75.22 (13.76) | 65.30 (24.93) | 1.244 | 0.214 |
| **Angry to calm** | 88.44 (10.71) | 76.30 (26.34) | 1.599 | 0.110 |
| **Talking Teacher – Emotions** | | | | |
| **Sad to happy** | 100.00 (0.00) | 85.80 (31.75) | 0.000 | 1.000 |
| **Worried to relaxed** | 100.00 (0.00) | 80.40 (14.57) | 1.000 | 0.317 |
| **Lonely to sociable** | 100.00 (0.00) | 88.20 (17.49) | 1.000 | 0.317 |
| **Bored to excited** | 78.00 (31.11) | 67.40 (33.41) | 1.000 | 0.317 |
| **Angry to calm** | 100.00 (0.00) | 73.60 (42.34) | 1.000 | 0.317 |
| **Talking EA – Emotions** | | | | |
| **Sad to happy** | 83.00 (2.82) | 100.00 (0.00) | - | - |
| **Worried to relaxed** | 87.50 (17.67) | 61.67 (7.50) | - | - |
| **Lonely to sociable** | 84.50 (6.36) | 86.67 (23.09) | - | - |
| **Bored to excited** | 60.60 (16.26) | 45.66 (41.86) | - | - |
| **Angry to calm** | 92.50 (10.60) | 100.00 (0.00) | - | - |
| **Talking Classmates – Emotions** | | | | |
| **Sad to happy** | 87.00 (10.00) | 83.70 (29.80) | 0.140 | 0.889 |
| **Worried to relaxed** | 89.66 (10.18) | 87.40 (16.13) | 0.631 | 0.528 |
| **Lonely to sociable** | 88.88 (10.81) | 83.00 (23.46) | 0.771 | 0.441 |
| **Bored to excited** | 72.66 (18.23) | 74.20 (38.31) | 0.169 | 0.866 |
| **Angry to calm** | 87.11 (12.04) | 83.40 (22.78) | 0.593 | 0.553 |
| **Talking Family – Emotions** | | | | |
| **Sad to happy** | 78.28 (28.90) | 64.14 (45.00) | 0.943 | 0.345 |
| **Worried to relaxed** | 74.57 (29.55) | 61.85 (44.42) | 1.214 | 0.225 |
| **Lonely to sociable** | 78.71 (29.85) | 66.85 (38.63) | 1.483 | 0.138 |
| **Bored to excited** | 63.14 (30.96) | 62.00 (35.60) | 0.524 | 0.600 |
| **Angry to calm** | 78.85 (30.87) | 67.14 (41.64) | 1.214 | 0.225 |
| **Talking Someone else – Emotions** | | | | |
| **Sad to happy** | 91.67 (14.43) | 80.00 (20.91) | - | - |
| **Worried to relaxed** | 91.00 (15.58) | 87.80 (17.15) | - | - |
| **Lonely to sociable** | 91.00 (15.58) | 72.60 (41.87) | - | - |
| **Bored to excited** | 90.33 (16.743) | 47.80 (45.19) | - | - |
| **Angry to calm** | 90.33 (16.74) | 67.80 (42.60) | - | - |
| **Help – Emotions** | | | | |
| **Sad to happy** | 79.63 (22.26) | 69.00 (46.69) | 0.674 | 0.500 |
| **Worried to relaxed** | 86.13 (17.15) | 63.57 (45.49) | 0.000 | 1.000 |
| **Lonely to sociable** | 76.75 (24.15) | 75.66 (27.42) | 0.135 | 0.893 |
| **Bored to excited** | 69.13 (26.16) | 52.00 (31.64) | 0.552 | 0.581 |
| **Angry to calm** | 80.38 (25.26) | 69.50 (36.99) | 0.730 | 0.465 |
| **Note. * *p* = 0.05** | | | | |
