# Supplementary material for: Evaluating the feasibility, fidelity, and preliminary effectiveness of a school-based intervention to improve the school participation and feelings of connectedness of elementary school students on the autism spectrum
Source: PLoS One. 2022 Jun 1;17(6):e0269098. doi: 10.1371/journal.pone.0269098 (PMC9159612; doi:10.1371/journal.pone.0269098)
Supplement: S6 Table — (DOCX) [file pone.0269098.s006.docx]

**S6 Table. Difference SSBS, SEI-E, belonging scale scores pre-post intervention, autism sample**

|  | **Pre**  **Mean (SD)** | **Post**  **Mean (SD)** | **Z score** | **P value** |
| --- | --- | --- | --- | --- |
| **SSBS-2** | | | | |
| Peer relations | 38.60 (8.784) | 38.20 (10.696) | 0.459 | 0.646 |
| Self-management/ compliance | 34.60 (5.873) | 33.50 (7.012) | 0.831 | 0.406 |
| Academic behaviour | 26.00 (6.00) | 26.50 (6.996) | 0.536 | 0.592 |
| Social competence total | 99.20 (18.683) | 98.20 (21.358) | 0.663 | 0.507 |
| Hostile/ irritable | 25.30 (7.775) | 26.10 (7.951) | 0.409 | 0.682 |
| Antisocial/ aggressive | 16.00 (6.464) | 16.00 (5.249) | 0.238 | 0.812 |
| Defiant/ disruptive | 14.80 (4.849) | 16.20 (5.922) | 1.191 | 0.234 |
| Antisocial behaviour total | 56.10 (18.181) | 58.30 (17.069) | 0.969 | 0.333 |
| **SEI-E** | | | | |
| Teacher student relationship | 37.44 (8.457) | 35.44 (11.326) | 0.000 | 1.000 |
| Peer support for learning | 22.67 (5.657) | 22.67 (6.325) | 0.566 | 0.571 |
| Family support for learning | 18.00 (2.121) | 17.67 (3.000) | 0.000 | 1.000 |
| Future goals and aspirations | 19.78 (5.449) | 17.89 (7.061) | 1.261 | 0.207 |
| Intrinsic motivation | 6.56 (3.046) | 6.67 (2.915) | 0.106 | 0.915 |
| Behavioural engagement | 7.78 (4.086) | 8.75 (3.655) | 0.106 | 0.916 |
| Disaffection | 8.78 (4.324) | 9.56 (4.246) | 0.430 | 0.667 |
| SEI-E total | 104.44 (18.487) | 100.33 (26.782) | 0.141 | 0.888 |
| **Belonging scale** | 28.30 (3.945) | 28.10 (6.297) | 0.153 | 0.878 |
| Note. SSBS-2: School Social Behaviour Scale-2; SEI-E: Student Engagement Instrument-Elementary. *p<0.05 | | | | |
