# Supplementary material for: Evaluating the feasibility, fidelity, and preliminary effectiveness of a school-based intervention to improve the school participation and feelings of connectedness of elementary school students on the autism spectrum
Source: PLoS One. 2022 Jun 1;17(6):e0269098. doi: 10.1371/journal.pone.0269098 (PMC9159612; doi:10.1371/journal.pone.0269098)
Supplement: S7 Table — (DOCX) [file pone.0269098.s007.docx]

**S7 Table. Difference between autism and TD scores in HCSBS, SEI-E, Belonging, In My Shoes scales pre-post In My Shoes intervention**

| **Measure** | **Students with autism** | | **Classmates** | |  |
| --- | --- | --- | --- | --- | --- |
|  | **Pre Mean (SD)** | **Post Mean (SD)** | **Pre Mean (SD)** | **Post Mean (SD)** | **p** |
| **HCSBS (n=27)** | | | | | |
| Peer relations | 48.50 (11.48) | 53.67 (10.89) | 71.74 (7.97) | 71.22 (11.31) | 0.145 |
| Self-management/ compliance | 42.30 (11.99) | 48.11 (9.99) | 59.30 (7.53) | 61.39 (8.41) | 0.106 |
| Social competence Total | 90.80 (21.49) | 101.78 (19.09) | 131.04 (14.46) | 132.61 (18.67) | 0.085 |
| Defiant/ Disruptive | 40.30 (15.28) | 39.11 (11.42) | 30.35 (9.80) | 25.39 (5.73) | 0.705 |
| Antisocial/ Aggressive | 32.80 (15.17) | 31.56 (10.35) | 24.70 (6.17) | 22.83 (6.07) | 0.781 |
| Antisocial Behaviour Total | 73.10 (29.72) | 70.67 (20.40) | 55.04 (15.25) | 47.22 (10.16) | 0.743 |
| **SEI-E (n=200)** | | | | | |
| Teacher student relationship | 37.44 (8.46) | 35.44 (11.33) | 36.15 (5.79) | 36.60 (6.21) | 0.718 |
| Peer support for learning | 22.67 (5.66) | 22.67 (6.33) | 23.66 (4.13) | 24.12 (4.08) | 0.852 |
| Family support for learning | 18.00 (2.12) | 17.67 (3.00) | 17.74 (2.42) | 17.70 (2.61) | 0.960 |
| Future goals and aspirations | 19.78 (5.45) | 17.89 (7.06) | 20.74 (3.65) | 21.17 (3.09) | 0.130 |
| Intrinsic motivation | 6.56 (3.05) | 6.67 (2.92) | 6.75 (3.20) | 8.89 (1.93) | 0.090 |
| Behavioural engagement | 7.78 (4.09) | 8.75 (3.66) | 9.20 (2.25) | 9.25 (2.13) | 0.813 |
| Disaffection | 8.88 (4.32) | 9.56 (4.25) | 8.89 (2.89) | 8.89 (2.61) | 0.963 |
| SEI-E total | 104.44 (18.49) | 100.33 (26.78) | 105.04 (13.66) | 108.49 (12.62) | 0.350 |
| **Belonging Scale (n=200)** | 28.30 (3.95) | 28.10 (6.29) | 30.22 (4.14) | 30.12 (4.49) | 0.815 |
| **In My Shoes (n=200)** | | | | | |
| Situation based | 13.50 (4.12) | 13.78 (3.63) | 15.76 (1.89) | 16.17 (1.89) | 0.590 |
| In the past week | 30.20 (12.10) | 33.78 (9.20) | 37.35 (5.28) | 36.37 (5.60) | 0.094 |
| Involvement | 18.000(4.37) | 17.89 (5.39) | 20.90 (3.59) | 20.43 (2.68) | 0.371 |
| Learning about the autism spectrum | 7.83 (0.41) | 7.83 (0.41) | 7.18 (1.27) | 7.50 (1.19) | 0.278 |
| *Note.* HCSBS, Home Community Social Behaviour Scale; SEI-E, Student Engagement Instrument – Elementary Version; *p<0.05; **p<0.01; p<0.001 | | | | | |
