# Supplementary material for: Evaluating the feasibility, fidelity, and preliminary effectiveness of a school-based intervention to improve the school participation and feelings of connectedness of elementary school students on the autism spectrum
Source: PLoS One. 2022 Jun 1;17(6):e0269098. doi: 10.1371/journal.pone.0269098 (PMC9159612; doi:10.1371/journal.pone.0269098)
Supplement: Attachment — Submitted filename: PLOS ONE review.docx [file pone.0269098.s008.docx]

Thank you for the opportunity to review Evaluating the feasibility, fidelity, and preliminary effectiveness of a school-based intervention to improve the school participation and feelings of connectedness of elementary school students on the autism spectrum submitted to the Research Article section of PLOS ONE. My comments are below:

You refer to students with autism spectrum disorder (ASD) a few different ways throughout the manuscript. It would help for clarity to simply refer to them as students with ASD as it’s the most current terminology.

I’m confused about the purpose of the *In my Shoes* curriculum. It states that it’s an online support for teachers and peers, but reads like a social skill training program for students with ASD. Perhaps offer more explanation of how the teachers or peers use the curriculum so as to not confuse the reader.

I don’t see how a training curriculum for teachers and peers builds connectedness for students with ASD. There’s needs to be a strong rationale for how this curriculum can do this, as well as break down connectedness into its component parts and create a rationale for sections of the curriculum targeting each component.

What was the purpose of the qualitative data collection? Was it to better understand answers on the survey, were only certain participants interviewed based on survey data or were they all interview? This question applies to the teachers, parents, and students with ASD as well. What were the procedures for selection and what was the intent beyond gathering more information. Was it to better understand scores on the assessment? Were participants with specific scores targeted for follow up or did it occur for everyone?

Its concerning that there’s an emphasis on school connectedness in the opening and there is no mention of connectedness in the methods section. Why wasn’t a measure of school connectedness included as a part of the battery of instruments used? If the measure used for school engagement and belonging is the proxy measure for school connectedness that needs to be made clear, but also should be defined in the opening section as the author’s definition of school connectedness.
