# Supplementary material for: Evaluating the feasibility, fidelity, and preliminary effectiveness of a school-based intervention to improve the school participation and feelings of connectedness of elementary school students on the autism spectrum
Source: PLoS One. 2022 Jun 1;17(6):e0269098. doi: 10.1371/journal.pone.0269098 (PMC9159612; doi:10.1371/journal.pone.0269098)
Supplement: Attachment — Submitted filename: Response to Reviewers_13.3.2022_R1Final.docx [file pone.0269098.s009.docx]

**Response to Reviewers**

Thank-you for reviewing our manuscript, ‘Evaluating the feasibility, fidelity, and preliminary effectiveness of a school-based intervention to improve the school participation and feelings of connectedness of elementary school students on the autism spectrum’. We have responded to comments and revised the manuscript accordingly. We have listed reviewer comments and our response and have cross referenced line numbers where the changes were affected in the marked manuscript. Revisions following specific reviewer recommendations are shown using track changes in the attached manuscript.

| **#** | **Reviewer Comment** | **Author Response** |
| --- | --- | --- |
| 1.1 | You refer to students with autism spectrum disorder (ASD) a few different ways throughout the manuscript. It would help for clarity to simply refer to them as students with ASD as it’s the most current terminology. | Language use related to autism in the manuscript reflects recent contributions to the literature characterising the language preferences of individuals on the autism spectrum (1-3). While educators, researchers and clinicians tend to prefer person-first language (e.g., student with autism; 4), autistic adults and other members of the autism community prefer identity first language (e.g., autistic student; 5). The language used in resulting intervention resources have been adapted to reflect the preferences of stakeholders in which they are intended. For example, the intervention manual uses person-first language as it was developed for use by educators; however, educators are encouraged to discuss language preferences with their students and use this at school. Whereas, the documentary style video included in the whole class program, which shares the school experiences of autistic students, uses identity first language in line with preferences of the autistic community. For the purposes of this manuscript, we have chosen to consistently use terminology ‘student/children on the autism spectrum’ and ‘autism’. It is recognised there is no absolute consensus, and our understanding of language preferences is continually evolving. However, we believe language is a powerful means for shaping how people view autism and, therefore, we have made an effort in the manuscript to avoid ableist language (i.e., disorder) so that researchers, educators and the broader community may become more accepting and accommodating of autistic individuals (1). |
| 1.2 | I’m confused about the purpose of the *In My Shoes* curriculum. It states that it’s an online support for teachers and peers, but reads like a social skill training program for students with ASD. Perhaps offer more explanation of how the teachers or peers use the curriculum so as to not confuse the reader. | We have made changes in the manuscript to more clearly articulate the intended outcomes and components of *In My Shoes* on pages 6 and 7 (see below for intended outcomes and components for ease of reference). We have also made reference in the manuscript to another recently published paper, which outlines, in more detail, the conceptualisation of the intervention, and the content and delivery of each intervention component. This paper includes a detailed schematic overview of whole class lesson plan topics.  *Intended outcomes:*   a. increase understanding and awareness of differences in the way students experience autism and school *(i.e., preferences);* b. increase feelings of being accepted, respected, included and supported by others in the school social environment *(i.e., school connectedness);* c. increase self-awareness of strengths and differences and the strengths and differences of peers *(i.e., sense of self);* d. improve confidence in their abilities to recognise when someone needs help, how to help others and ask for help at school *(i.e., sense of self and activity competence)*; and e. improve students’ interpersonal empathy and use of pro-social behaviours to include peers in the classroom and playground *(i.e., activity competence).*   *Components:*   1) standardised online professional learning and ongoing face-to-face or online support for teachers and school leadership staff; 2) teacher-led whole class lesson plans linked to Australian health curriculum in schools; 3) peer training for selected peers; 4) activity ideas to incorporate key messages across the whole school; and 5) weekly parent information handouts and invitations for parents to participate in the intervention. |
| 1.3 | I don’t see how a training curriculum for teachers and peers builds connectedness for students with ASD. There’s needs to be a strong rationale for how this curriculum can do this, as well as break down connectedness into its component parts and create a rationale for sections of the curriculum targeting each component. | While *In My Shoes* includes a professional learning component (which encompasses all intervention outcomes; see discussion point 2) and a peer training component (which focuses on supporting selected peers to further build on their interpersonal empathy and use of prosocial behaviours to support students in the classroom and playground), the main component of the intervention is the whole class program. As per previous comment we have more clearly articulated the intended outcomes and components (i.e., professional learning, peer training, whole class program, whole school activity ideas and parent involvement) of *In My Shoes* in the manuscript on pages 6 and 7. We have also included additional information about the content of the whole class program and how it addresses intervention outcomes. We have also made reference to a recently published paper, which provides detailed information on the content of each intervention component and rationale for how it addresses intervention outcomes. |
| 1.4 | What was the purpose of the qualitative data collection? Was it to better understand answers on the survey, were only certain participants interviewed based on survey data or were they all interview? This question applies to the teachers, parents, and students with ASD as well. What were the procedures for selection and what was the intent beyond gathering more information. Was it to better understand scores on the assessment? Were participants with specific scores targeted for follow up or did it occur for everyone? | As outlined on page 17, qualitative interviews were conducted with *all* teachers, parents and students on the autism spectrum that participated in the study. The interviews had several purposes including to verify and enrich quantitative data and gather more information about the feasibility, fidelity and perceived benefits of the intervention from different participants perspectives. For example, teachers were asked questions like ‘How easy was it to implement *In My Shoes* in your classroom?’, and ‘Do you think peers have experienced any benefits as a result of participating in *In My Shoes*? If so, can you please share some specific examples with me?’. These lines of questioning enabled us to understand the subjective experience of participants that were not captured quantitatively.  It was not feasible to include all students (i.e., typically developing peers) in interviews, however, this will be considered in future research such as pilot studies or RCT’s. We have added this as a limitation to the study on page 42 and 43, stating ‘typically developing peers were not included in qualitative interviews as it was not deemed feasible for this study, however, will be considered in future pilot studies or RCTs’. |
| 1.5 | Its concerning that there’s an emphasis on school connectedness in the opening and there is no mention of connectedness in the methods section. Why wasn’t a measure of school connectedness included as a part of the battery of instruments used? If the measure used for school engagement and belonging is the proxy measure for school connectedness that needs to be made clear, but also should be defined in the opening section as the author’s definition of school connectedness. | In the first sentence of the introduction we make reference to various terms used over the years to describe the concept of school connectedness, including school belonging, bonding, engagement and attachment. We chose to use the subheading ‘subjective school experiences’ in the methods section to outline measures we used to understand students sense of connectedness. This included Experience Sampling Method to explore the nature and quality of students on the autism spectrum experience while participating at school and school engagement and belonging measures with all students participating in the study called the Student Engagement Instrument – Elementary Version and the Belonging Scale (outlined on page 11 to 14 of the manuscript). We have added a sentence on page 12 and 13 to explain this to the reader. |
| 2.1 | The abstract was lacking in important details. It needed a clear rationale statement. The age or grade of the sample needed to be identified. The sample description is inadequate. It is not clear whether the 10 students were students with autism. We subsequently learn that data was also collected from about 200 “typically developing “ children, but this is not mentioned or detailed in the abstract. The “preliminary evidence” that is referred to needs to be given some content. | We have added a clear aim to the abstract and included details regarding the sample as suggested. We have also provided more detail relating to study findings. Please refer to these changes on page 2. |
| 2.2 | The introduction needed a more thorough conceptual or theoretical understanding of belonging and school connectedness in particular. This conceptual understanding is important to provide the reader with a sense of the intervention being implemented. The authors could also have reviewed the literature more extensively on attempts to promote school connectedness, even if these interventions were not targeted specifically at children on the spectrum. I subsequently note that in the method the authors refer to quite extensive research that they have conducted prior to the development of the intervention. A more extensive review of some of this research needed to part of the rationale of the study. | Authors of this study have previously conducted a systematic review evaluating the psychometric properties of school connectedness measures to help inform use of measures in the current study. In this systematic review we provide a detailed overview of the concept of school connectedness. We have now made reference to this systematic review, including examples of affective, cognitive and behavioural factors that were found to contribute towards students sense of school connectedness. These changes can be found on page 3.  We have now provided specific examples of existing school-based interventions to highlight the paucity of interventions available that immerse all students in learning that aims to improve students’ interpersonal empathy and ability to display behaviours that help others participate and feel included at school. These changes can be found on page 4.  We have also made reference to a recently published paper that provides more detail about the development of *In My Shoes* from conceptualisation to implementation in the school environment which can be found on page 5. This paper describes how literature on autism, school participation and intervention research were integrated to develop a theoretical model of school participation. It also describes the iterative process of developing the intervention using focus groups, a Delphi study, online surveys, and reference group consultation.  Rather than adding to what is an already lengthy paper, we have decided to draw reference to existing papers that explain the conceptualisation of the intervention for the reader. |
| 2.3 | Participant recruitment, characteristics and numbers were inadequately detailed in the participant section . We need to know more about the numbers and sample and how the schools and students were selected and recruitment rates (if possible) etc. A particular source of frustration were the lack of details in this section about the recruitment and characteristics of the “typically developing” students. Initially on reading this method section I wondered why the peers were not sampled as part of the outcome, as they formed such an important part of the rationale? Later there is a suggestion that measures were given to non autistic children but this is not presented in the sample characteristics in the method section. We learn in the results and the supplementary table that 200 student surveys were completed? Who are these students? How were they recruited and why are we not provided with any participant characteristics in this regard? Were they part of the informed consent procedure? | Eligibility criteria to participate in the study is included in the participant section (page 5 and 6), however, details about sample characteristics are described in the results section under ‘recruitment capability and sample characteristics’ from page 19. Details regarding how schools were recruited is detailed from line 205.  It was not feasible to collect demographic information on all 200 typically developing students participating in the study and therefore these details are not reported. Typically developing students were asked to complete a battery of measures with students on the autism spectrum to evaluate changes in outcomes pre-post intervention. These results are reported in Table 4 (page 34 and 35) and in supplementary tables.  We have added detail to the procedures and methods section to more clearly outline process around informed consent with typically developing students. These changes can be found on page 8. |
| 2.4 | Rationale for the screening measures needed to be more clearly articulated eg. The teacher efficacy scale. | Screening assessments were conducted pre-intervention to identify and describe the skills and abilities of students on the autism spectrum and to confirm their eligibility for the study as described on page 9. We have added additional rationale for using the teacher self-efficacy scale, which can be found on page 10. |
| 2.5 | The questions around “feasibility” seemed more akin to questions around process evaluations and acceptability rather than feasibility - this construct needs to be more clearly defined and operationalised. | We have included definitions for feasibility and fidelity on page 5. We were guided by Orsmond & Cohn (6) and Bowen et al. (7) when designing this feasibility study. Components of feasibility evaluated in this study (e.g., recruitment capability and sample characteristics, data collection procedures and outcome measures, appropriateness, implementation and practicality) listed on page 5, are based on objectives and guiding questions outlined in Orsmond & Cohn’s (6) paper. |
| 2.6 | There was some great creativity in some of the measures used such as ESM technology. However there generally needed to be a clearer link between program rationale and expected outcomes and the measures | We have made several changes in the proximal outcome measures section to more clearly link measures that we used to intervention outcomes. These changes can be found from page 11 to 17. |
| 2.7 | Many measures had no sample items- it would have helped the reader to get sample items on all measures . | Example sample items are included for relevant measures as requested which can be found between pages 9 and 17. |
| 2.8 | The behavioural observation measures were interesting- what steps however were taken by the rater/s to avoid the likelihood of confirmation bias? | We have added a sentence to explain steps that were taken to minimise confirmation bias for behavioural observations, which can be found on page 12. |
| 2.9 | With regard to the results -the tables generally needed to contain the Ns- whether it be with the teachers, students on the spectrum, the “typically developing” students. As indicated above the sample characteristics of the typically developing sample needed to be detailed. It was difficult to work out at times who the student participants were and their number for each of the measures and analyses. It is only when you dig into the supplementary material that you get a sense of the wider evaluation and comparison with the typically developing students. It is unfortunate that this was not presented more clearly. | Sample sizes in relevant tables have been added for clarity for the reader, which can be found on pages 19 to 21 and 34 to 35. |
| 2.10 | The discussion was relatively solid although it was at times difficult to determine the accuracy of the interpretation because the sample and results were not well presented. | As per previous comments, we have provided additional details regarding the sample and provided sample sizes in tables as suggested. We have also expanded in the limitations section regarding the impact of the small sample which can be found on page 42. |
| 2.11 | The limitations needed to be expanded upon. In particular the small sample size limitation was understated. There were so many measures with such a small sample that the notion of type 1 error needed to be discussed. Other limitations such as the lack of a comparison group in pre and post measures also needed to be stated more strongly – even if this is a preliminary study. Do thesese pre and post outcome measures reflect any reliable or or meaningful change | We have added more detail to the limitations section and highlighted that while this study evaluated the preliminary effectiveness of the intervention, the purpose of the study, first and foremost, was to evaluate the feasibility of the intervention in the school environment and therefore a small sample size was appropriate to determine whether larger scale studies are warranted (see page 42). |
| 3.1 | There is little description of the previous literature or the theoretical underpinning of the In My Shoes program. I believe the paper would be improved if these two areas were addressed | Please refer to response to reviewer comment 2.2. |
| 3.2 | I think that some careful editing of the Procedures and Measures section would be beneficial. | The procedures and measures section has been reviewed and edited where appropriate. |

**References**

1.	Bottema-Beutel K, Kapp SK, Lester JN, Sasson NJ, Hand BN. Avoiding ableist language: Suggestions for autism researchers. Autism in Adulthood. 2021;3(1):18-29.

2.	Botha M, Hanlon J, Williams GL. Does language matter? Identity-first versus person-first langauge use in autism research: A response to Vivanti. Journal of Autism & Developmental Disorders. 2021.

3.	Vivanti G. Ask the editor: What is the most appropriate way to talk about individuals with a diagnosis of autism? Journal of Autism & Developmental Disorders. 2020;50(2):691-3.

4.	Hodges A, Cordier R, Joosten A, Bourke-Taylor H. Expert consensus on the development of a school-based intervention to improve the school participation and connectedness of elementary students on the autism spectrum: A Delphi study. Focus on Autism and Other Developmental Disabilities. 2021:1-11.

5.	Bury SM, Jellett R, Spoor JR, Hedley D. "It defines who I am" or "It's something I have": What language do [Autistic] Australian adults [on the autism spectrum] prefer? Journal of Autism and Developmental Disorders. 2020.

6.	Orsmond GI, Cohn ES. The distinctive features of a feasibility study: objectives and guiding questions. OTJR: Occupation, Participation and Health. 2015:1-9.

7.	Bowen DJ, Kreuter M, Spring B, Cofta-Woerpel L, Linnan L, Weiner D, et al. How we design feasibility studies. American Journal of Preventative Medicine. 2009;36(5):452-7.
